# Impact of Heavy Metal and Resistance Genes on Antimicrobial Resistance: Ecological and Public Health Implications

**DOI:** 10.3390/genes16060625

**Published:** 2025-05-24

**Authors:** Carlos G. Sánchez-Corona, Luis Uriel Gonzalez-Avila, Cecilia Hernández-Cortez, Jorge Rojas-Vargas, Graciela Castro-Escarpulli, Hugo G. Castelán-Sánchez

**Affiliations:** 1 Laboratorio de Investigación Clínica y Ambiental, Departamento de Microbiología, Escuela Nacional de Ciencias Biológicas, Instituto Politécnico Nacional, Prolongación de Carpio y Plan de Ayala s/n, Col. Casco de Santo Tomas, Miguel Hidalgo, Ciudad de Mexico 11340, Mexico; gabrielwaldorf.cc@gmail.com (C.G.S.-C.); u_gza@hotmail.com (L.U.G.-A.); 2 Laboratorio de Bioquímica Microbiana, Departamento de Microbiología, Escuela Nacional de Ciencias Biológicas, Instituto Politécnico Nacional, Prolongación de Carpio y Plan de Ayala s/n, Col. Casco de Santo Tomas, Miguel Hidalgo, Ciudad de Mexico 11340, Mexico; cecihercor@hotmail.com; 3 Departments of Biology and Microbiology & Immunology, Western University, London, ON N6A 3K7, Canada; jarvar86@gmail.com; 4 Department of Pathology and Laboratory Medicine, Western University, London, ON N6A 3K7, Canada

**Keywords:** heavy metal resistance, heavy metal resistome, co-selection of resistance

## Abstract

Heavy metals (HMs) are widespread pollutants that can exert selection pressure on microbial populations due to their toxicity and persistence, leading to the evolution of heavy metal resistance genes (HMRGs). These genes are part of the resistome, and their spread often occurs via mobile genetic elements that allow co-selection with antibiotic and biocide resistance genes. Such processes have an impact on microbial biodiversity, biogeochemical cycling and public health in agriculture, industry and urban areas. The selection pressure exerted by HM promotes the spread of multidrug-resistant strains and thus increases ecological and health risks. This review discusses the interaction between HMRGs and genetic determinants such as virulence genes that influence biofilm formation, cellular homeostasis and oxidative stress. It also discusses the dual role of HMRGs in promoting ecological functions such as bioremediation while potentially limiting them by reducing microbial diversity. Understanding such interactions contributes significantly to targeting different systems to overcome the challenges associated with antimicrobial resistance (AMR).

## 1. Introduction

There is an important relationship that exists between heavy metals and microorganisms. It influences the evolutionary dynamics of ecosystems and is also one of the most important issues related to environmental aspects and public health. The ecological relevance arises from the ubiquity of the heavy metal, whereby it selects microbial communities and promotes their resistance mechanisms for the occurrence of mutations and horizontal gene transfer [1].

Chemical speciation, i.e., the chemical forms or states of HMs in the environment, controls mobility, persistence and toxicity. These processes are regulated by physicochemical factors such as pH, redox potential and organic material and influence the uptake into an organism and the toxic effects they can cause [2]. Selection pressure from HMs has driven the evolution of a range of microbial resistance mechanisms, from active excretion of metals using efflux pumps to conversion to less harmful chemical forms using specific enzymatic processes. Most of these mechanisms are mediated by genes specialized for metal resistance, mostly operons, e.g., *mer*, *ars*, *chr*, *cad* and *pbr*, which encode transport proteins, detoxification proteins and regulatory proteins [3,4]. Furthermore, HMRGs not only ensure the survival of microbes in polluted environments but are also associated with the resistance of microbes to other antimicrobial agents such as antibiotics and biocides through co-selection mechanisms. The transfer of HMRGs between microbial species can not only increase the prevalence of resistance but also increase the pathogenicity and adaptability of microorganisms to hostile environments [5,6]. In addition, HMs and HMRGs are involved in the disruption of important ecological processes, such as the reduction of microbial biodiversity in soils, affecting the decomposition of organic matter and the recycling of essential nutrients [7], and altering the ecological balance in aquatic environments, promoting phenomena such as eutrophication and affecting the health of aquatic biota and, ultimately, humans through bioaccumulation in food chains [7,8].

Against this background, the present work presents an interdisciplinary analysis of the role of HMRGs in microbial resistance and homeostasis in relation to public health and global ecosystem dynamics. Our aim is to contribute to strategies for the ecologically sustainable management of pollution and AMR through a comprehensive analysis of the genetic and biochemical mechanisms of resistance.

### 1.1. Heavy Metals: Speciation and Bioavailability

Heavy metals (HMs) are an ambiguous group of elements that include some transition metals, metalloids, lanthanides and actinides with different physicochemical properties but which are considered ubiquitous agents in the environmental context, either as part of the Earth’s crust or as a product of anthropogenic activities that exert selective pressure on microorganisms and influence their genetic variation mechanisms [1].

HMs have different chemical forms or states in the environment that depend on physicochemical factors such as temperature, pH or redox potential and on the interaction with other compounds such as organic material. These chemical forms determine their bioavailability, toxicity, persistence and mobility, which are also regulated by mechanical, physicochemical and biological migration mechanisms, which, in turn, depend on factors such as fluid dynamics, gravity, metabolism and trophic networks [9].

The chemical speciation of HMs generalizes their presence and increases their availability for absorption, use in essential physiological functions or to cause toxic effects on microorganisms. At low concentrations, metals such as zinc (Zn) and copper (Cu) act as enzymatic cofactors and are involved in the regulation of antioxidant functions; others such as cadmium (Cd) and mercury (Hg), even at low concentrations, can interfere with protein synthesis and enzymatic function, with negative effects on viability and growth capacity [2].

In the atmosphere, HMs occur as gasses (e.g., elemental mercury and methylarsenes), particles and aerosols, mainly from industrial and vehicle emissions. Their persistence and dispersion depend on reactions such as oxidation and photooxidation, as well as climatic and topographical factors, and generally ends with their deposition (dry or wet) in water bodies, agricultural soils, forest soils, etc. [10,11].

The speciation and mobility of HMs in soil is determined by soil composition, with the most active components being clay minerals, organic matter, microorganisms and their derived complexes. HMs can occur as solid precipitates, adsorption complexes or free ions in solutions resulting from processes influenced by redox potential (Eh) and pH, e.g., adsorption–desorption, oxidation–reduction, methylation–demethylation or chelation [12]. In vegetation, dissolved HMs such as chromium (Cr), lead (Pb), arsenic (As), nickel (Ni), Cd and Hg are transported by diffusion or convection into the rhizosphere, where they are taken up by the roots and then bioaccumulated from their entry into the cellular level through phosphate transport channels or by diffusion through aquaporins [13,14]. The action of rainwater or irrigation can mobilize HMs, either as soil-bound particles or in their dissolved form, which can lead to their accumulation in groundwater [10].

In lentic and lotic waters, HMs can be found on the surface or in bottom sediments in dissolved or suspended form, bound to colloids or to organic and inorganic complexes. The speciation of HMs and the physicochemical conditions of the environment determine their bioavailability, so that they can enter aquatic biota through sorption or absorption processes. This leads to the bioaccumulation of HMs in these organisms and increases their concentration at higher trophic levels [7,15].

### 1.2. Heavy Metal Resistance

HMs have multiple effects on microorganisms, which can be both toxic and adaptive, depending on the concentration, type of metal and exposure time. Some of these mechanisms include substitution by metal–ligand bonds affecting the biological function of target molecules; the generation of reactive oxygen species (ROS) by covalent and ionic reduction–oxidation (redox) reactions of metal ions with cellular thiols or by Fenton-type reactions with transition metals (iron (Fe), Cu and Ni); disruption of ion regulation in specific membrane transporters and effects on DNA composition [16,17].

The cellular stress exerted by HMs on microorganisms has promoted the evolution of genes that regulate the expression of adaptive proteins that enable resistance to these toxic compounds. Common mechanisms include efflux and sequestration systems that remove metals from the cell or encapsulate them in safe compartments, as well as transformation into more tolerable forms [18,19].

### 1.3. Heavy Metal Resistome

The resistome was originally described on the basis of clinical studies on human pathogens and is defined as the totality of genes present in genomes or mobilomes that confer direct (phenotypic resistance genes) and indirect (silent and proto-resistance genes) resistance to a specific group of antimicrobial agents in a given microorganism at a given time point [20]. This collection of genes is the result of chromosomal mutations and gene transfers that have taken place over millions of years and have been influenced by various biotic and abiotic factors [21]. HM resistance and the corresponding resistome are considered one of the oldest and most widespread microbial adaptation strategies, as shown by their wide phylogenetic distribution in the Archaea, Bacteria and Eukarya domains [22,23]. They are also thought to have evolved around 2.4 billion years ago after the Great Oxidation Event, when heavy metals became bioavailable [24,25].

**Figure 1:**
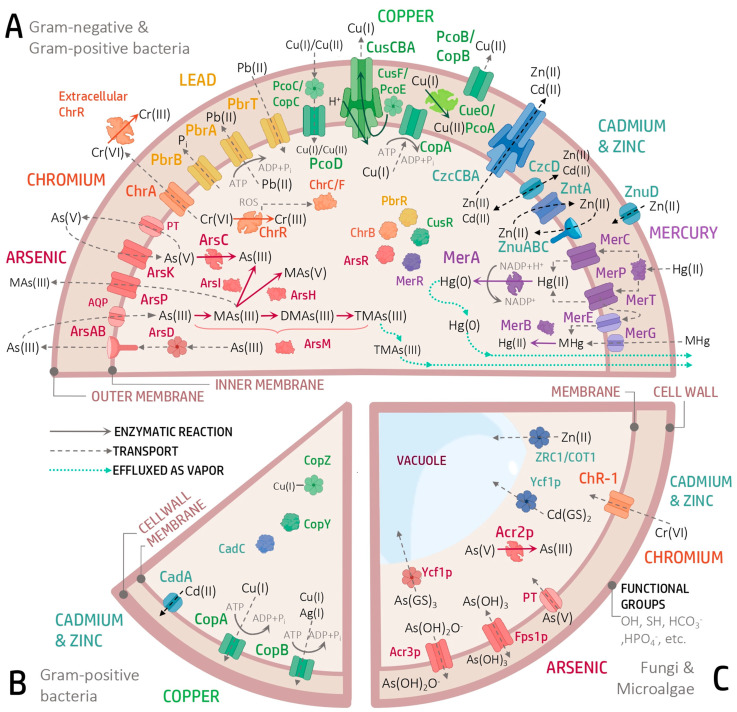
Heavy metals resistance systems for prokaryotes and eukaryotes. (**A**) The resistance systems to As, Cr, Pb and Hg correspond to both Gram-negative and Gram-positive organisms and reside in the *ars*, *chr*, *pbr* and *mer* operons, respectively, while the system for Cu includes the *cue*, *cus*, *pco* and *cop* operons. The resistance systems exclusively for Gram-negative bacteria to Zn include the *znu* and *zur* operons, in addition to the ZnT proteins and the *czc* system, which is shared with Co and Cd. (**B**) For Gram-positive bacteria, the systems for Cu and Cd exclusively include homologs of *cop* and the *cad* operon, respectively. (**C**) Eukaryotic systems include fungal membrane proteins resistant to As, Cr, Pb, Cd and Zn and vacuolar transporters for Cd, Zn and As, as well as reactive compounds in the cytoplasm and functional groups in the cell wall of fungi and microalgae that react with HMs. Figure created from BioRender.

The heavy metal resistome consists of genes encoding proteins responsible for the detoxification, transformation or exclusion of a specific metal, located mainly in the cell membrane and cytoplasm (Figure 1). Other reactions mediated by non-specialized proteins also take place in other cellular environments [26].

## 2. Non-Specific Systems

### 2.1. Extracellular Medium

In this environment, metals are susceptible to conversion and/or adsorption by reaction with excreted metabolites or products of extracellular enzymes. For example, Pb^2+^ can form metal–ligand complexes with the siderophores pyochelin and pyoverdine, which are chelators that are excreted as intermediates in Fe uptake [27]. Cr^6+^ can be reduced to Cr^3+^ by H_2_S produced by sulfide adenosyl transferase and adenylyl sulfate reductase, as well as by Fe^2+^ generated by Fe^3+^-reducing microorganisms [28]. On the other hand, Hg^2+^ can be adsorbed by sulfide or organosulfur compounds [4]. These interactions are even more prominent when metals react with exopolysaccharides (EPS), a group of carbohydrate polymers of wide compositional diversity and with a predominant anionic charge, which are present free in the extracellular medium or deposited on the cell wall surface [29,30]. Furthermore, EPS are related to biofilm development in bacteria, fungi, cyanobacteria and microalgae, playing a crucial role in cell adhesion, cohesion, aggregation, nutrient bioavailability and protection from the external environment, which is relevant for the cellular resistance system [31,32,33,34,35].

### 2.2. Cell Wall

Compositional differences in the cell wall of bacteria, fungi, algae and other microorganisms can be translated into various proportions of functional groups, such as amide (-CO-NR_2_), amine (-NH_2_), carboxyl (-COOH), carbonyl (C=O) and hydroxyl (-OH). These functional groups can establish covalent wall–metal bonds or exchange ions with cations or anions of wall compounds, such as EPS [16]. This results in a higher metal accumulation capacity in Gram-positive bacteria compared to Gram-negative bacteria due to their higher proportion of peptidoglycan and the presence of anionic compounds such as teichoic acid and teichuronic acid [36].

### 2.3. Cell Membrane

Metal entry into cells is adventitious and occurs primarily through competition with essential compounds that are structurally or reactively similar, using transmembrane transport pathways. For example, Cr enters through sulfate permeases [37], while As is transported by phosphate transporters and via aquaglyceroporins [38]. Pb and Cd enter cells through divalent metal transporters, such as Mn and Zn, or through Fe transport-specific ligands, such as siderophores [39,40]. Cr compounds, in contrast, predominate as insoluble complexes that are inaccessible to the cell. However, transient products of extracellular Cr^6+^ reduction, such as hydroxyl complexes or NAD^+^-Cr^3+^, can enter the cell through mechanisms that have not yet been clarified [41,42].

In fungi, metal entry is also carried out by transporters or by passive diffusion due to the high permeability that the membrane can present in some species [43]. In algae, metal transport is not completely understood, but it is believed to also be based on sulfate, phosphate and divalent ion transporters [44].

### 2.4. Cytoplasm

In this region, interactions focus on the intracellular access of metals through active transport in the membrane and their subsequent compartmental accumulation in organelles, with the vacuole being the main center of cellular homeostasis in eukaryotes [45,46]. The effectiveness of this organelle is due, in part, to vacuolar ATPases (V-ATPase), which translocate protons and act as Ca^2+^/H^+^ exchangers in the vacuolar membrane. This maintains a proton gradient that allows HM sequestration [47,48,49]. The vacuole also hosts a variety of proteases and peptidases, including serine carboxypeptidases involved in the synthesis of phytochelatins [50,51]. These are cysteine-rich polypeptides that can form complexes with HMs due to the partial negative charge of the sulfhydryl groups of these residues. This metal sequestration mechanism, based on a high sulfur content, is also present in glutathione (GSH), a tripeptide composed of glutamate, cysteine and glycine, related to the control of oxidative stress, as well as in metallothioneins, cysteine-rich proteins with specific binding to metals (Cd, Pb, Zn and Cu), responsible for the transport of metal ions from the cytosol to the organelles [52,53]. Some cyanobacteria have demonstrated the ability to synthesize metallothioneins and siderophores, Fe-chelating compounds [54].

## 3. Specific Systems

### 3.1. Arsenic

The *ars* operon (*arsRDABC* or *arsRBC*) comprises the genes responsible for the detoxification of As and is found in both plasmids and bacterial chromosomes (Figure 1A). As can enter the cells as arsenate (AsO_4_^3−^) or arsenite (AsO_3_^3−^) via phosphate or glycerol transporters. The efflux of arsenite (As^3+^) occurs via the ArsAB complex or directly by ArsB, a membrane-bound efflux pump that can function alone, although its activity is enhanced by ArsA, an intracellular ATPase. In other cases, ArsB can be replaced or supplemented by Acr3, a functional homolog found in bacteria and fungi. ArsK is another transporter that removes metals such as antimony (Sb^3+^) and bismuth (Bi^3+^) [48,55]. In the cytosol, ArsC, an arsenate reductase, converts arsenate (As^5+^) into arsenite (As^3+^), a less toxic form. This process involves a redox cascade mediated by thiolate nucleophiles. ArsC is coupled to glutaredoxin in species such as *Staphylococcus aureus* and *Bacillus subtilis* or to thioredoxin in *Escherichia coli* (plasmid R773). In eukaryotes (Figure 1C) such as *Saccharomyces cerevisiae*, the Acr2p reductase fulfills similar functions for As^5+^ [56]. Arsenite is removed from the cytoplasm by being exported by the permease Acr3p or transported into the vacuole by the ABC transporter Ycf1p as glutathione conjugates [As(GS)_3_], which also functions for complexes with Cd [Cd(GS)_2_]. The aquaglyceroporin Fps1p can control the influx and efflux of As^3+^ [55].

The methyltransferase ArsM can convert As^3+^ into methylarsenite (MAs^3+^), which can be effluxed through ArsP, ArsK or channels such as GlpF. In the second reaction, ArsM produces dimethylarsenite (DMAs^3+^) and, finally, trimethylarsenite (TMAs^3+^), a non-toxic and volatile compound. Under aerobic conditions, these products are rapidly oxidized to non-toxic pentavalent forms [26]. ArsI, an extradiol dioxygenase capable of cleaving the C–As bond, demethylates MAs^3+^ to As^3+^ and other trivalent aromatic arsenic compounds, such as Rox(III) (roxarsone), Nit(III) (nitarsone) and p-ASA(III) (p-arsanilic acid) (Figure 1A) [57].

In addition, ArsD (Figure 1A), a polypeptide chaperone, transfers arsenite to ArsA, while ArsR acts as a negative regulatory repressor and represses basal and maximal expression of the operon [58,59].

Other related operons are *aio*, which is involved in arsenite oxidation, and *arr*, which is involved in anaerobic arsenate respiration. The *aio* operon (*aioSRABcytC* or *aioRSABC*) encodes the arsenite oxidase AioAB, the histidine kinase AioS, the transcriptional regulator AioR and the As^3+^-binding protein AioX [60]. The *arr* operon (*arrSRABD*) contains the ArrAB protein, which belongs to the dimethyl sulfoxide reductase family and acts as the final electron acceptor by transferring electrons from the respiratory chain to arsenate [61].

### 3.2. Chromium

The *chr* operon, which comprises the genes involved in Cr resistance (Figure 1A), is found in both plasmids and chromosomes of diverse organisms, including Eukarya, Bacteria and Archaea. This operon encodes proteins such as ChrA, a chromate (Cr^6+^) efflux pump belonging to the CHR superfamily, and ChrB, a chromate-sensitive transcriptional regulator. In addition, the proteins ChrC and ChrF, which correspond to Fe and manganese superoxide dismutases (Fe-SOD and Mn-SOD), participate in the reduction of ROS generated during Cr detoxification [15,62]. ChrA is responsible for extruding chromate out of the cell, while, in some organisms, such as fungi of the phylum Ascomycota, its homolog CHR-1 transports chromate into the cytosol, facilitating its intracellular accumulation [41] (Figure 1C). An additional key mechanism for Cr detoxification involves the reduction of Cr^6+^ to Cr^3+^, catalyzed by diverse chromate reductases that vary in their mechanism of action and cofactors (Figure 1A). The main enzymes involved are:

- ChrR/YieF/NemA: Flavoproteins that reduce chromate and its intermediates simultaneously. ChrR transfers one or two electrons from NADH, forming Cr^5+^ or Cr^4+^, which are then converted to Cr^3+^. During this process, ROS can be generated, but ChrR also exhibits activity that protects the cell against these reactive species [63]. YieF reduces Cr^6+^ directly to Cr^3+^, generating less ROS, while NemA uses NADPH as an electron donor to reduce nitroaromatic compounds and Cr^6+^ [64].
- NfsA/NfsB: Flavoproteins that transfer electrons to reduce nitrogen compounds and quinones and are also involved in the reduction of Cr^6+^ [65].
- NfoR: This enzyme reduces Cr^6+^ indirectly through the reduction of FMN. This non-catalytic mechanism can be enhanced in the presence of Cu^2+^ [66].

Other enzymes, such as glutathione reductase (GRd), dihydrolipoamide dehydrogenase (LpDH) and C-type cytochromes in sulfate-reducing bacteria, also participate in the reduction of Cr^6+^ by electron transfer, generating H_2_O_2_, which subsequently reacts to form hydroxyl radicals, thus completing the detoxification process [67].

### 3.3. Lead 

*Cupriavidus metallidurans* CH34 is a model organism from which several genes related to Pb detoxification have been identified, including three *pbr* loci, which encode Pb^2+^ response regulators: PbrR, PbrR2 (previously PbrR691) and PbrR3 (previously PbrR710). In addition, the *pbrUTRABCD* operon, which expression depends on PbrR and the divergent pbr promoter, acts as a repressor of the transcription of opposing structural genes [68].

Regarding the proteins encoded in *pbrUTRABCD* (Figure 1A), they include PbrT, a transmembrane permease responsible for Pb^2+^ uptake; PbrA, a P-type ATPase that transports Zn^2+^, Cd^2+^ and Pb^2+^ from the cytoplasm to the periplasmic space; PbrB, an undecaprenyl pyrophosphate phosphatase, which function is to release phosphate and, when combined with Pb^2+^, forms a precipitate in the periplasmic space; PbrC, an aspartic peptidase that could act as a signal peptidase for the PbrB precursor; PbrD, a high-affinity metallochaperone for Pb^2+^, rich in cysteine, serine and proline and finally, PbrU, which function is still unknown and has been found exclusively in *C. metallidurans* CH34 [35,58,60]. Pb^2+^ efflux can be mediated by homologous proteins associated with the transport of other divalent ions such as Cd^2+^ and Zn^2+^, including CadA, ZntA and PbrA, P-type ATPases that transport Pb ions against the concentration gradient using ATP hydrolysis as an energy source [69,70]. The regulation of these transporters includes genes such as *pbrR*, *zntR*, *cadR*, *cueR* and *merR*, belonging to the Mer family, initially identified in Hg^2+^ resistance systems [70].

### 3.4. Copper 

Several Cu resistance systems have been described (Figure 1A), some of which are encoded in chromosomes. One of these is the *cue* system, composed of the genes *cueR* and *cueO*. CueR is a regulator of cueO and copA expression and is homologous to MerR. CueO is a periplasmic multicopper oxidase that converts Cu^1+^ to Cu^2+^ and reduces O_2_ to water in an electron transfer process. Another system is *cus* (*cusCFBA*), which consists of the proteins CusA, CusB, CusC and CusF. CusCBA is an efflux complex for silver (Ag) and Cu that uses the proton gradient across the inner membrane as an energy source to expel excess of these metals into the extracellular space. CusA, a trimeric protein of the Resistance-Nodulation-Division (RND) family, is in the inner membrane and facilitates the efflux of Cu^1+^ and Ag^1+^. CusB, a soluble periplasmic protein of the Membrane Fusion Protein (MFP) family, stabilizes the CusCBA complex and, due to its structural flexibility and substrate-binding capacity, could act as a switch of the complex function. CusF is a periplasmic metallochaperone that transfers Cu to CusCBA via CusB, thereby improving detoxification efficiency [71,72].

In contrast, other Cu efflux systems are found in plasmids, such as *pcoABCDRSE* and *copABCDRS* (Figure 1A), two homologous systems identified in *E. coli* isolated from pigs fed Cu-supplemented diets and in *Pseudomonas syringae* from tomato plants exposed to Cu-based fungicides, respectively [73]. These systems consist mainly of four structural genes (*pcoABCD/copABCD*) and the regulators PcoRS/CopRS. PcoA/CopA are soluble periplasmic proteins homologous to multicopper oxidases such as CueO. PcoB/CopB are localized in the outer membrane and facilitate Cu import. CopC/PcoC, located in the periplasm functions as chaperones that bind Cu and transfer it to PcoD/CopD, present in the inner membrane and responsible for Cu transport. PcoRS/CopRS form a two-component regulatory system, where PcoS/CopS is a membrane-associated sensor and PcoR/CopR is a transcriptional activator. The *pco* system also includes PcoE, a periplasmic protein that transports Cu to PcoB, which is highly inducible by Cu, although not essential for the functioning of the system [12,72].

In Gram-positive bacteria such as *Enterococcus hirae* and *Lactococcus lactis* (Figure 1B), the *cop* system includes *copA*, *copB*, *copY* and *copZ*. CopA captures Cu for the metalation of a cuproenzyme at the cytoplasmic membrane, while CopB effluxes excess Cu^+1^ and Ag^+1^. CopY encodes a Cu-inducible transcriptional repressor, and CopZ is a Cu chaperone [74,75].

### 3.5. Cadmium 

The bacterial resistance system to Cd is mainly based on the *cad* and *czc* operons. The *cad* operon (Figure 1B), identified in Gram-positive bacteria such as *S. aureus*, includes CadA, an efflux pump belonging to the P-type ATPase superfamily, and CadC, a regulator of the ArsR/SmtB family, together with CadR, a transcriptional regulator of the MerR family. CadA transports Cd^2+^ into the periplasm and is regulated by CadR, which has a high affinity for Cd^2+^, as well as a relative affinity for Zn^2+^ and Pb^2+^. Other proteins of the operon include the transporter CadD, the regulator CadX and the carbonic anhydrase CadW, which can sequester Cd^2+^ [76,77].

The *czc* operon (Figure 1A), identified in Gram-negative bacteria such as *Pseudomonas aeruginosa*, consists of up to eight genes. Some of these encode the cation-proton transmembrane transporter CzcCBA, the inducible two-component system CzcRS and the transporter CzcD, which belongs to the Cation Diffusion Facilitator (CDF) protein family. The CzcCBA complex includes CzcA, an RND protein in the inner membrane; CzcC, which is in the outer membrane and could modify the specificity of the complex to include sensitivity to Cd and cobalt (Co) and CzcB, a membrane fusion protein responsible for expelling excess ions from the cytoplasm and periplasm to the outside of the cell [78,79]. The CzcRS system is composed of the sensor histidine kinase CzcS and the response regulator CzcR, which acts through a phosphorylation cascade. Other genes in the operon, such as *czcI* and *czcN*, are being studied to determine their functions and products [80].

### 3.6. Zinc 

Intracellular regulation of the Zn levels is mediated by several molecular systems, mainly transporters (Figure 1A). Zn entry and exit in bacterial cells is regulated by high- and low-affinity uptake systems, depending on intracellular Zn levels. The ZnuABC complex, consisting of ZnuA, ZnuB and ZnuC, belongs to the ATP-binding cassette transporter family and acts as a high-affinity Zn uptake regulatory system. In this complex, ZnuA is a Zn-binding protein located in the periplasmic space, while ZnuB, located in the inner membrane, interacts with ZnuA, facilitating Zn transport across the cytoplasmic membrane. ZnuC, an ABC-type ATPase, drives active Zn transport into the cytoplasm by ATP hydrolysis [81]. In bacteria such as *Neisseria meningitidis*, this system has been associated with ZnuD, an extracellular Zn transport protein located in the outer membrane [55].

In addition to transporters, Zn homeostasis is also maintained by uptake regulators such as Zur, a transcriptional regulator responsive to reversible Zn binding, which belongs to the FUR (Ferric Uptake Regulator) superfamily of metal-sensitive transcriptional regulators. Zur proteins possess high-affinity and extremely sensitive binding sites, capable of detecting intracellular Zn concentrations in the femtomolar (fM) range. This mechanism allows a gradual response in gene expression to fluctuations in Zn levels, reflected in the successive formation of oligomers and multimers as the concentration increases. Zur has been reported in Gram-negative bacteria such as *E. coli* and Gram-positive bacteria such as *B. subtilis* [82]. In the latter, the import of Zn mediated by PfeT or ZosA, a P1B-type ATPase (like *copA* and *cadA*), which expression is induced by H_2_O_2_ and Fe, has also been documented, with proposed functions in protection against oxidative stress and Fe efflux [78].

ZnT proteins, which belong to the CDF family, constitute a group of transporters widely distributed in prokaryotes and eukaryotes. In bacteria, several of these have been identified, such as YiiP, a Zn^2+^/H^+^ antiporter; ZitB, which promotes Zn^2+^ efflux across the cytoplasmic membrane; ZntA, a P-ATPase that transports Zn^2+^; ZntB, a Zn^2+^/H^+^ symporter and CzcD, a Cd^2+^, Co^2+^ and Zn^2+^/H^+^-K^+^ antiporter, which is involved in maintaining intracellular divalent cation and potassium homeostasis [83] (Figure 1A). In yeasts such as *S. cerevisiae*, ZRC1 and COT1 (vacuolar ZnTs) have been identified, which act as Zn^2+^/H^+^ antiporters and regulate Zn^2+^ homeostasis, transporting and storing it in the vacuole [84] (Figure 1C).

### 3.7. Mercury 

The Hg resistance system is based on the *mer* operon, which has diverse structural configurations and contains several coding genes (Figure 1A), The most common and well known are *merA*, *merT*, *merP* and *merR*. This mechanism begins with the specific uptake of Hg. For inorganic Hg, MerP, a receptor protein located in the periplasm, captures and transfers the Hg^2+^ ion to MerF or MerT. MerF facilitates the entry of Hg^2+^ into the cytosol as a transmembrane carrier protein. Organic Hg, on the other hand, enters through the proteins MerG and MerE, located in the periplasmic and inner membrane, respectively. MerT, located in the inner membrane, is a transport protein for organic and inorganic Hg with three transmembrane domains. Other identified transporters include MerC, which transports organic and inorganic Hg, and MerH, which transports mercuric ions (Hg_2_^2+^), both crossing the inner membrane [3,4].

Once in the cytosol, MerA, a mercury(II) reductase that uses NADPH as an electron source, transforms Hg^2+^ into Hg^0^, which is released from the cell as vapor. MerB, an organomercurial lyase, demethylates organic Hg to generate Hg^2+^, which is processed by MerA [85].

The regulation of the *mer* operon is carried out by MerR, an activator and repressor protein dependent on Hg^2+^ (narrow spectrum) and, occasionally, organomercurial compounds (broad spectrum). Furthermore, MerD is a MerR antagonist protein that negatively regulates the expression of operon genes by binding to the MerO operator region, controlling the positive and negative expression of *mer* genes [86].

## 4. Heavy Metals Homeostasis, Resistance and Virulence

Of the metals reviewed here, Zn, Ni, Cr, Cu and Co can be considered essential micronutrients for cellular functions and components of relevant proteins such as RNA and DNA polymerases, urease, cytochrome and cytochrome c-oxidase. Although they are manifestly toxic at high concentrations, they are considered toxic elements with metabolic relevance [87]. Faced with this potential toxicity, bacteria seek to maintain the homeostasis of these metal ions—that is, a delicate balance in their intracellular levels that are sufficient to guarantee their functions while avoiding intoxication [88]. These mechanisms not only allow survival in hostile environments but are also linked to the regulation of resistance and virulence genes [89].

### Co-Selection of Resistance

The presence of multiple antimicrobial agents in the environment, in addition to HMs—for example, antibiotics, polycyclic aromatic hydrocarbons (PAHs), microplastics, nanoparticles and residues from disinfectants and personal care products—facilitates the selection and spread of common resistance genes, either due to similarities between molecular resistance mechanisms or due to the presence of genes expressing different resistance phenotypes in the same mobile genetic element (MGE), such as plasmids, transposons and integrons, facilitating their mobilization through horizontal gene transfer (HGT), which involves the exchange of genes between genomes of the same or different microbial species [87,90,91]. This process, called co-selection, includes different models such as co-resistance, since the selective pressure of at least one of these antimicrobial agents is sufficient to cause the transfer of the entire MGE, as the resistance genes are physically linked; cross-resistance, where the molecular pathways of action of the different antimicrobial agents converge towards a common target, stimulating resistance to both agents in a single event and co-regulation, as these genes can respond to the same regulator or set of regulators, allowing an organism to coordinate its response to different environmental stressors together [92,93,94].

Other transfer mechanisms include transformation, where the presence of stressors, such as HMs, can stimulate the direct incorporation of extracellular DNA, including HMRGs, and transduction, where lysogenic phages can transfer HMRGs from the viral genome to infected bacteria, enhancing viral host resistance against HMs toxicity [67].

The presence of HMRGs in MGEs is broad and widespread, and other resistance determinants such as virulence genes or antimicrobial resistance genes (ARGs) for antibiotics or biocides are also commonly found (Table 1). In *E. coli*, the Tn21 transposon contains the *mer* operon, while, in *Acidithiobacillus caldus*, it contains the *ars* operon [95,96]; plasmids such as pMOL30, which confer resistance to Cd, Zn and Co, have been reported in *C. metallidurans* CH34 [97] and HMRGs such as *chrA* have been found in class 1 integrons (intI1–dfrA12–orf–aadA2–qacEΔ1–sul1–chrA–padR) in *Aeromonas aquatica* MX16A [98]. On the other hand, recent research has pointed to HMs resistance through transposable elements in eukaryotes such as the fungus *Paecilomyces variotii*, where the putative transposon Hφ (HEPHAESTUS) was identified, which participates in tolerance to metals such as Cu, Zn and Pb [99].

## 5. Ecological Homeostasis

At the ecosystem level, antimicrobial resistance (AMR) is a fundamental phenomenon for genetic evolution and microbial adaptation. Many of the genes carried by MGEs exhibit parasitic, beneficial and cooperative expression traits, which are relevant in microbial ecological dynamics [105].

**Figure 2:**
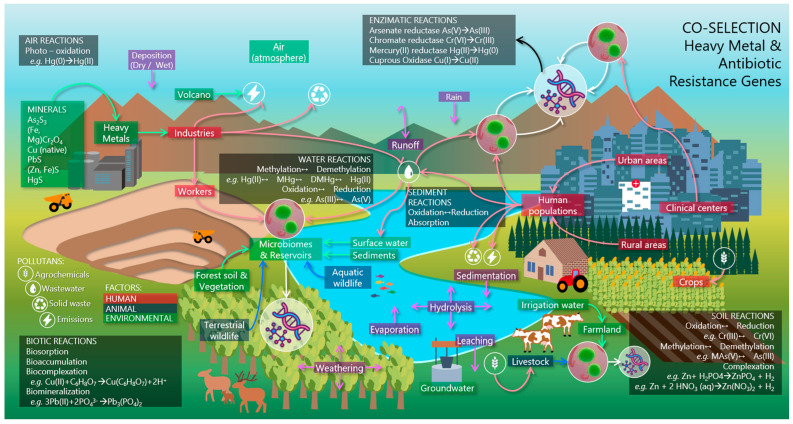
Development and dissemination of HMRGs in natural and anthropogenic environments, influenced by the environmental chemical speciation of the HMs and by the presence of other sources of pollution (wastewater, solid waste, atmospheric emissions from combustion or energy consumption and agrochemicals). Three main factors are recognized: a human factor, which includes human populations and anthropogenic environments such as agricultural, industrial areas and clinical centers; an animal factor, which includes wild and farmed organisms and an environmental factor, made up of natural resources and microbial reservoirs, such as human and animal microbiota, and the microbiomes of soil and water bodies. It is in these reservoirs where HMRGs mainly reside and where AMR is promoted. Figure created from WPS Slides.

ARGs, including HMRGs, can be considered global-reaching factors that coexist with inducing antimicrobial agents. On the one hand, the co-selective pressure of multiple antimicrobial agents can produce hysteresis, allowing resistance mechanisms and their dissemination to persist even after their elimination [106]. On the other hand, ARGs have a high capacity for mobilization and encoding across phylogenetically distant species, thus surpassing the reach capacity of antimicrobial-resistant bacteria (ARBs) [94]. This phenomenon is directly linked to human activities and can affect the microbiomes of natural or anthropogenic ecosystems (Figure 2) [34].

Microbiomes present in different environmental settings play an essential role in biogeochemical cycles, ecosystem health and interaction with higher organisms. They are complex and dynamic communities containing diverse microorganisms, including bacteria, archaea, fungi, microalgae, protozoa and viruses [5].

### 5.1. Natural Environments

#### 5.1.1. Soils

Soil bacteria such as *Burkholderia* spp., *Pseudomonas* spp., *Streptomyces* spp. and some species of *Rhizobium* spp. stand out for their ability to tolerate high concentrations of HMs and possess HMRGs, such as efflux pumps (*czc* for Zn) and detoxification systems (*cop* and *cus* for Cu). These mechanisms not only allow the survival of these bacteria in toxic environments but also contribute to their role in the decomposition of organic matter, promotion of plant growth and the carbon cycle, allowing symbiotic interactions to be established between fungi, (e.g., *Trichoderma* spp. and *Mycorrhizae* spp.) and plants to improve nutrient uptake [107]. Furthermore, the study of soil microbiota using multi-omics approaches has shown that HMRGs are also associated with a decrease in microbial biodiversity, as they favor the proliferation of resistant strains while sensitive ones are displaced. This can lead to a loss of ecological functions critical to soil health, such as the degradation of organic compounds and the recycling of essential nutrients, which can further be aggravated by the presence of other contaminants, such as antibiotics and pesticides [108,109,110].

#### 5.1.2. Plants

The interaction between soil bacteria, plants and HMRGs has significant effects on ecosystems. Plant growth-promoting bacteria (PGPRs), such as *Pseudomonas* spp., *Bacillus* spp. and *Rhizobium* spp., are crucial for nutrient acquisition and the production and secretion of auxins, gibberellins and cytokinins (plant growth-promoting substances); phytohormone production; nutrient solubilization (PO^4−^, Fe^2+^ and Fe^3+^); metabolic stimulation of roots; increased disease resistance and reduced toxicity of HMs, thus reducing plant stress and facilitating plant growth in contaminated soils [111,112,113].

#### 5.1.3. Wildlife

The microbiota of wild animals generally does not contain ARBs, given the isolation from their hosts; however, they can also interact with human populations or impacted sites, motivating the uptake of ARBs and ARGs (including HMRGs) and subsequently their short- and long-distance spread, through local interactions or migratory movements, respectively [114]. Honeybees and their microbiota are an example of this dissemination, acting as ARB vectors and as a link between natural and anthropic environments, as they are affected by the use of pesticides and antibiotics for prolonged periods [115].

#### 5.1.4. Water Bodies and Aquatic Wildlife

In the current global context, many water bodies act as concentration centers for wastewater of diverse compositions, including HMs and other antimicrobials, as well as ARGs and HMRGs [108]. The contact of aquatic organisms with contaminated water can affect the health of many other organisms through the food chain, including people [8,87].

The proliferation of ARBs can also alter the ecological balance of aquatic bodies, enhancing processes such as eutrophication, where the excessive growth of algae and bacteria reduces dissolved oxygen, affecting aquatic life, degrading water quality [91,109] and altering the native microbiome, which, in freshwater bodies, is dominated by *Cyanobacteria* spp., *Betaproteobacteria* spp. and *Bacteroidetes* spp., while saltwater environments are rich in *Prochlorococcus* spp. and *Pelagibacter* spp., microorganisms essential for the global carbon cycle [116].

In groundwater, microbial diversity is less abundant and diverse than in other environments; therefore, the introduction of antimicrobial agents or ARGs can alter this diversity and generate dangerous metabolites [17,117]. It is also a potential source of contamination of drinking water and contributes to the development of antibiotic resistance in humans, increasing virulence, pathogenicity and the incidence of outbreaks of bacterial infections [118].

### 5.2. Anthropic Environments

#### 5.2.1. Agricultural Environments

In agriculture and livestock farming, the use of compounds containing HMs (Cu, Cr and Zn) is common, as components of medicines, growth promoters and additives in livestock feed, although some, such as As, present in compounds such as Rox(III), Nit(III) and *p*-ASA(III), are being abandoned because they are not considered safe [119].

In any case, the use of these compounds and practices such as the application of livestock manure as fertilizer facilitate the generation and dissemination of HMRGs in environmental microbiomes [120,121] and can modify the enzymatic activity of the microbiota of agricultural soils, where the genera *Bacillus* spp. and *Pseudomonas* spp. Predominate, and *Nitrosomonas* spp., which are involved in processes such as nitrogen fixation and the degradation of xenobiotic compounds [122].

Other practices with the same effect in plant agriculture are irrigation with wastewater and the application of fertilizers and pesticides in agricultural soils, which introduce metals such as Cu, Cd and Pb, which can generate a potential risk to the health of consumers by promoting their bioaccumulation in livestock tissues, milk, eggs and other by-products [98].

#### 5.2.2. Industrial Environments

Industrial activities, such as mining, metallurgy and inappropriate waste management, are significant sources of HMs pollution in soil, water and air. These activities introduce high concentrations of metals, which exert selective pressure on local microbial communities and contribute to the mobility of resistance genes in microbial communities. These dynamics pose significant challenges in resistance management in the environment and public health due to the persistence and mobility of these genes in the environment [123,124]. The microbiomes of industrial environments where HMs predominate include highly resistant species, including bacteria from the phyla Proteobacteria, Acidobacteria, Firmicutes and Bacteroidetes and fungi from the phyla Ascomycota and Ciliophora, capable of not only resisting toxic metals but also contributing to biogeochemical cycles, such as the degradation of contaminants [125,126].

#### 5.2.3. Hospital Environments

Hospitals represent a significant reservoir of microorganisms, including multidrug-resistant pathogens such as *S. aureus*, *Acinetobacter baumannii*, *Klebsiella pneumoniae* and *P. aeruginosa*, many of them carrying antibiotic and HMRGs [127]. The intensive use of antibiotics and disinfectants, hospital waste management and sanitation strategies exert selective pressures on microorganisms present on surfaces, medical devices and hospital wastewater, allowing the survival of resistant strains and promoting the co-selection of resistance genes, since some genetic mechanisms can confer resistance to both HMs and antibiotics and biocides in bacteria such as *A. baumannii* and *P. aeruginosa*, facilitating dispersion in the environment through the wastewater flow [5,6].

Several studies have linked exposure to HMs with the selection of ARBs. However, epidemiological studies examining this relationship are scarce. For example, in 2020, Bazzi et al. [128] reported that multidrug-resistant *A. baumannii* (MDRAb) was the main pathogen isolated from wounded US soldiers during the Iraq War. In these conflict areas, high levels of HMs (Hg, Zn, Cu, Ni, Pb and Cr) were detected in soil and debris, which may have favored resistance to HMs and antibiotics, contributing to severe infections in field hospitals. Likewise, in 2021, Eggers et al. [129] found that, in a human population from urban areas with high HM pollution (Wisconsin, USA), elevated urinary Pb levels correlated with a higher prevalence of ARBs, including methicillin-resistant *S. aureus* (MRSA), vancomycin-resistant enterococci (VRE), fluoroquinolone-resistant Gram-negative bacilli (RGNB) and *Clostridium difficile*.

## 6. Conclusions

HMRGs are a key component of microbial resistance and homeostasis. They play a fundamental role in the adaptation of microorganisms to environments with toxic concentrations of HMs and in the stabilization of essential metabolic functions. However, their importance goes beyond the microbial sphere, as they have a direct impact on the development and spread of AMR, especially for antibiotics.

The relationship between HMRGs and ARGs is complex and multilayered. It is linked by processes such as co-selection, which not only facilitates HGT between bacteria, including pathogens, but also enhances the ability of microorganisms to adapt and survive in different ecosystems. In clinical settings, this phenomenon contributes to the emergence of multidrug-resistant bacteria such as *P. aeruginosa* and *S. aureus*, which poses a major public health challenge by limiting available therapeutic options and increasing the burden of infectious diseases.

From an ecological perspective, HMRGs also affect natural dynamics by promoting changes in microbial biodiversity and altering critical functions in biogeochemical cycles, which can also have negative consequences for agricultural productivity and water quality. In addition, the spread of HMRGs in natural and anthropogenic environments may facilitate the transfer of resistance to previously unexposed microbial reservoirs, increasing the risk of genetic pollution on a global scale.

In the long term, the persistence and spread of HMRGs could exacerbate the problems associated with AMR and worsen their impact on human, animal and environmental health. This phenomenon underscores the importance of pursuing integrated approaches such as the One Health perspective that take into account the link between natural ecosystems, production systems and public health. In addition, concerted efforts are needed to monitor the presence and mobility of HMRGs, develop strategies to mitigate their impact and promote sustainable practices that minimize the release of heavy metals and antibiotics into the environment.

HMRGs are critical microbial adaptation factors that simultaneously pose an emerging threat to public health and environmental sustainability. Addressing this problem requires coordinated action that combines scientific research, environmental management and public policy. The aim is to reduce the associated risks and ensure the resilience of ecosystems in the face of current and future global challenges.

## Figures and Tables

**Table 1:** Examples of MGEs with HMRGs and ARGs.

Bacteria	MGEs	HMRGs	ARGs	References
*Staphylococcus aureus*	*pPA3*	CadR (Cd) As	*ermR* (erythromycin) *tetR* (tetracycline) *pCH11* (bacteriocine)	[100]
*Escherichia fergusonii*	*p280_40A*	Te, Cu, Hg and Ag operons	*APH(4)-Ia* (hygromycin B) *AAC(3)-Iva* (apramycin) *strA* (streptomycin) *straB* (streptomycin) *sul1* (sulfonamide) *sul2* (sulfonamide) *bla_CTX–M-2_* (β-lactamase) *tetA* (tetracycline) *tetB* (tetracycline)	[101]
*Listeria* spp.	*pLM80*	cadA2C2 (Cd)	*bcrABC* (benzalkonium chloride) *tmr* (crystal violet and malachite green)	[102]
*Shigella flexneri*	Tn21	*mer* operon (Hg)	*aadA1* (streptomycin and spectinomycin)	[95]
*Salmonella enterica*	*IncFIB*	*mer* operon (Hg)	*qacEdelta1* (antiseptics)	[103]
*Pseudomonas aeruginosa* BJ86	*pBJ86*	*mer* operon (Hg)	*bla*_DIM−1_ (β-lactamase) *qnrVC6* (fluoroquinolone) *mexCD−oprJ* (fluoroquinolones, tetracyclines and chloramphenicol)	[104]

## References

[1] Margaryan A., Panosyan H., Birkeland N.-K. (2021). Heavy Metal Resistance in Prokaryotes: Mechanism and Application. Microorganisms for Sustainability.

[2] He Z., Shen J., Li Q., Yang Y., Zhang D., Pan X. (2023). Bacterial Metal(Loid) Resistance Genes (MRGs) and Their Variation and Application in Environment: A Review. Sci. Total Environ..

[3] Naguib M.M., El-Gendy A.O., Khairalla A.S. (2018). Microbial Diversity of Mer Operon Genes and Their Potential Rules in Mercury Bioremediation and Resistance. Open Biotechnol. J..

[4] Priyadarshanee M., Chatterjee S., Rath S., Dash H.R., Das S. (2022). Cellular and Genetic Mechanism of Bacterial Mercury Resistance and Their Role in Biogeochemistry and Bioremediation. J. Hazard. Mater..

[5] Berendonk T.U., Manaia C.M., Merlin C., Fatta-Kassinos D., Cytryn E., Walsh F., Bürgmann H., Sørum H., Norström M., Pons M.-N. (2015). Tackling Antibiotic Resistance: The Environmental Framework. Nat. Rev. Microbiol..

[6] Zhang S., Huang J., Zhao Z., Cao Y., Li B. (2020). Hospital Wastewater as a Reservoir for Antibiotic Resistance Genes: A Meta-Analysis. Front. Public Health.

[7] Squadrone S. (2020). Water Environments: Metal-Tolerant and Antibiotic-Resistant Bacteria. Environ. Monit. Assess..

[8] Zhao Y., Xu R., Cox S.F., Qiao M., Guo H. (2024). Metals Are Overlooked in the Evolution of Antibiotic Resistance. Soil Ecol. Lett..

[9] Deng H., Tu Y., Wang H., Wang Z., Li Y., Chai L., Zhang W., Lin Z. (2022). Environmental Behavior, Human Health Effect, and Pollution Control of Heavy Metal(Loid)s toward Full Life Cycle Processes. Eco -Environ. Health.

[10] Bishop K., Shanley J.B., Riscassi A., de Wit H.A., Eklöf K., Meng B., Mitchell C., Osterwalder S., Schuster P.F., Webster J. (2020). Recent Advances in Understanding and Measurement of Mercury in the Environment: Terrestrial Hg Cycling. Sci. Total Environ..

[11] Luo X., Bing H., Luo Z., Wang Y., Jin L. (2019). Impacts of Atmospheric Particulate Matter Pollution on Environmental Biogeochemistry of Trace Metals in Soil-Plant System: A Review. Environ. Pollut..

[12] Li P., Nayeri N., Górecki K., Becares E.R., Wang K., Mahato D.R., Andersson M., Abeyrathna S.S., Lindkvist-Petersson K., Meloni G. (2022). PcoB Is a Defense Outer Membrane Protein That Facilitates Cellular Uptake of Copper. Protein Sci..

[13] Al-Makishah N.H., Taleb M.A., Barakat M.A. (2020). Arsenic Bioaccumulation in Arsenic-Contaminated Soil: A Review. Chem. Pap..

[14] Kim R.-Y., Yoon J.-K., Kim T.-S., Yang J.E., Owens G., Kim K.-R. (2015). Bioavailability of Heavy Metals in Soils: Definitions and Practical Implementation—A Critical Review. Environ. Geochem. Health.

[15] Rahman Z., Singh V.P. (2019). The Relative Impact of Toxic Heavy Metals (THMs) (Arsenic (As), Cadmium (Cd), Chromium (Cr)(VI), Mercury (Hg), and Lead (Pb)) on the Total Environment: An Overview. Environ. Monit. Assess..

[16] Leong Y.K., Chang J.-S. (2020). Bioremediation of Heavy Metals Using Microalgae: Recent Advances and Mechanisms. Bioresour. Technol..

[17] Hemme C.L., Deng Y., Gentry T.J., Fields M.W., Wu L., Barua S., Barry K., Tringe S.G., Watson D.B., He Z. (2010). Metagenomic Insights into Evolution of a Heavy Metal-Contaminated Groundwater Microbial Community. ISME J..

[18] Baker-Austin C., Wright M.S., Stepanauskas R., McArthur J.V. (2006). Co-Selection of Antibiotic and Metal Resistance. Trends Microbiol..

[19] Pal A., Bhattacharjee S., Saha J., Sarkar M., Mandal P. (2022). Bacterial Survival Strategies and Responses under Heavy Metal Stress: A Comprehensive Overview. Crit. Rev. Microbiol..

[20] Wright G.D. (2010). The Antibiotic Resistome. Expert Opin. Drug Discov..

[21] Perry J.A., Westman E.L., Wright G.D. (2014). The Antibiotic Resistome: What’s New?. Curr. Opin. Microbiol..

[22] Banu H., Prasad K.P. (2017). Role of Plasmids in Microbiology. J. Aquac. Res. Dev..

[23] Biswas R., Halder U., Kabiraj A., Mondal A., Bandopadhyay R. (2021). Overview on the Role of Heavy Metals Tolerance on Developing Antibiotic Resistance in Both Gram-Negative and Gram-Positive Bacteria. Arch. Microbiol..

[24] Chen S.-C., Sun G.-X., Yan Y., Konstantinidis K.T., Zhang S.-Y., Deng Y., Li X.-M., Cui H.-L., Musat F., Popp D. (2020). The Great Oxidation Event Expanded the Genetic Repertoire of Arsenic Metabolism and Cycling. Proc. Natl. Acad. Sci. USA.

[25] Chi Fru E., Rodríguez N.P., Partin C.A., Lalonde S.V., Andersson P., Weiss D.J., El Albani A., Rodushkin I., Konhauser K.O. (2016). Cu Isotopes in Marine Black Shales Record the Great Oxidation Event. Proc. Natl. Acad. Sci. USA.

[26] Li L.-G., Xia Y., Zhang T. (2017). Co-Occurrence of Antibiotic and Metal Resistance Genes Revealed in Complete Genome Collection. ISME J..

[27] Tiquia-Arashiro S.M. (2018). Lead Absorption Mechanisms in Bacteria as Strategies for Lead Bioremediation. Appl. Microbiol. Biotechnol..

[28] Long D., Hashmi M.Z., Su X., Pongpiachan S. (2019). Cr(VI) Reduction by an Extracellular Polymeric Substance (EPS) Produced from a Strain of *Pseudochrobactrum saccharolyticum*. 3 Biotech.

[29] Islam S.T., Lam J.S. (2014). Synthesis of Bacterial Polysaccharides via the Wzx/Wzy-Dependent Pathway. Can. J. Microbiol..

[30] Osemwegie O.O., Adetunji C.O., Ayeni E.A., Adejobi O.I., Arise R.O., Nwonuma C.O., Oghenekaro A.O. (2020). Exopolysaccharides from Bacteria and Fungi: Current Status and Perspectives in Africa. Heliyon.

[31] Bhunia A., Lahiri D., Nag M., Upadhye V., Pandit S. (2022). Bacterial Biofilm Mediated Bioremediation of Hexavalent Chromium: A Review. Biocatal. Agric. Biotechnol..

[32] Kumawat T.K., Kumawat V., Sharma S., Kandwani N., Biyani M., Nadda A.K., Sajna K.V., Sharma S. (2021). Applications of EPS in Environmental Bioremediations. Microbial Exopolysaccharides as Novel and Significant Biomaterials.

[33] Morais M.G., Santos T.D., Moraes L., Vaz B.S., Morais E.G., Costa J.A.V. (2022). Exopolysaccharides from Microalgae: Production in a Biorefinery Framework and Potential Applications. Bioresour. Technol. Rep..

[34] Poole K. (2017). At the Nexus of Antibiotics and Metals: The Impact of Cu and Zn on Antibiotic Activity and Resistance. Trends Microbiol..

[35] Zayed A., Mansour M.K., Sedeek M.S., Habib M.H., Ulber R., Farag M.A. (2022). Rediscovering Bacterial Exopolysaccharides of Terrestrial and Marine Origins: Novel Insights on Their Distribution, Biosynthesis, Biotechnological Production, and Future Perspectives. Crit. Rev. Biotechnol..

[36] Pushkar B., Sevak P., Parab S., Nilkanth N. (2021). Chromium Pollution and Its Bioremediation Mechanisms in Bacteria: A Review. J. Environ. Manag..

[37] Ward N.P., DeNicola G.M. (2019). Sulfur Metabolism and Its Contribution to Malignancy. Int. Rev. Cell Mol. Biol..

[38] Mukhopadhyay R., Bhattacharjee H., Rosen B.P. (2014). Aquaglyceroporins: Generalized Metalloid Channels. Biochim. Biophys. Acta.

[39] Himeno S., Yanagiya T., Enomoto S., Kondo Y., Imura N. (2002). Cellular Cadmium Uptake Mediated by the Transport System for Manganese. Tohoku J. Exp. Med..

[40] Khan A., Singh P., Srivastava A. (2017). Synthesis, Nature and Utility of Universal Iron Chelator—Siderophore: A Review. Microbiol. Res..

[41] Gutiérrez-Corona J.F., Romo-Rodríguez P., Santos-Escobar F., Espino-Saldaña A.E., Hernández-Escoto H. (2016). Microbial Interactions with Chromium: Basic Biological Processes and Applications in Environmental Biotechnology. World J. Microbiol. Biotechnol..

[42] Puzon G.J., Roberts A.G., Kramer D.M., Xun L. (2005). Formation of Soluble Organo-Chromium(III) Complexes after Chromate Reduction in the Presence of Cellular Organics. Environ. Sci. Technol..

[43] Kumar V., Dwivedi S.K. (2019). Hexavalent Chromium Stress Response, Reduction Capability and Bioremediation Potential of *Trichoderma* sp. Isolated from Electroplating Wastewater. Ecotoxicol. Environ. Saf..

[44] Torelli A., Marieschi M., Castagnoli B., Zanni C., Gorbi G., Corradi M.G. (2008). Identification of S2-T A63: A CDNA Fragment Corresponding to a Gene Differentially Expressed in a Cr-Tolerant Strain of the Unicellular Green Alga *Scenedesmus acutus*. Aquat. Toxicol..

[45] Aufschnaiter A., Büttner S. (2019). The Vacuolar Shapes of Ageing: From Function to Morphology. Biochim. Biophys. Acta Mol. Cell Res..

[46] Wang J., Chen C. (2006). Biosorption of Heavy Metals by Saccharomyces Cerevisiae: A Review. Biotechnol. Adv..

[47] Lv Q., Yan L., Jiang Y. (2021). The Importance of Vacuolar Ion Homeostasis and Trafficking in Hyphal Development and Virulence in Candida Albicans. Front. Microbiol..

[48] Pivato M., Ballottari M. (2021). *Chlamydomonas reinhardtii* Cellular Compartments and Their Contribution to Intracellular Calcium Signalling. J. Exp. Bot..

[49] Vasanthakumar T., Rubinstein J.L. (2020). Structure and Roles of V-Type ATPases. Trends Biochem. Sci..

[50] Luxmi R., Blaby-Haas C., Kumar D., Rauniyar N., King S.M., Mains R.E., Eipper B.A. (2018). Proteases Shape the Chlamydomonas Secretome: Comparison to Classical Neuropeptide Processing Machinery. Proteomes.

[51] Parzych K.R., Klionsky D.J. (2019). Vacuolar Hydrolysis and Efflux: Current Knowledge and Unanswered Questions. Autophagy.

[52] Deshmukh M., Jangam S.S., Wankhede S.B. (2024). Potential Use of Microalgal Metallothioneins and Phytochelatins in Bioremediation. Microbiome-Assisted Bioremediation.

[53] Manikandan A., Suresh Babu P., Shyamalagowri S., Kamaraj M., Muthukumaran P., Aravind J. (2022). Emerging Role of Microalgae in Heavy Metal Bioremediation. J. Basic Microbiol..

[54] Majhi P., Nayak S., Samantaray S.M. (2021). Microalgal Bioremediation of Toxic Hexavalent Chromium: A Review. Environmental and Agricultural Microbiology: Applications for Sustainability.

[55] Yang H.-C., Fu H.-L., Lin Y.-F., Rosen B.P. (2012). Pathways of Arsenic Uptake and Efflux. Curr. Top. Membr..

[56] Kumari N., Jagadevan S. (2016). Genetic Identification of Arsenate Reductase and Arsenite Oxidase in Redox Transformations Carried out by Arsenic Metabolising Prokaryotes—A Comprehensive Review. Chemosphere.

[57] Pawitwar S.S., Nadar V.S., Kandegedara A., Stemmler T.L., Rosen B.P., Yoshinaga M. (2017). Biochemical Characterization of ArsI: A Novel C-as Lyase for Degradation of Environmental Organoarsenicals. Environ. Sci. Technol..

[58] Slyemi D., Bonnefoy V. (2012). How Prokaryotes Deal with Arsenic(†). Environ. Microbiol. Rep..

[59] Zhu Y.-G., Xue X.-M., Kappler A., Rosen B.P., Meharg A.A. (2017). Linking Genes to Microbial Biogeochemical Cycling: Lessons from Arsenic. Environ. Sci. Technol..

[60] Bhardwaj A. (2022). Understanding the Diversified Microbial Operon Framework Coupled to Arsenic Transformation and Expulsion. Biologia.

[61] Zhang J., Liu J., Zheng F., Yu M., Shabala S., Song W.-Y. (2022). Comparative Analysis of Arsenic Transport and Tolerance Mechanisms: Evolution from Prokaryote to Higher Plants. Cells.

[62] Branco R., Morais P.V. (2016). Two Superoxide Dismutases from TnOtchr Are Involved in Detoxification of Reactive Oxygen Species Induced by Chromate. BMC Microbiol..

[63] Deepa A., Mishra B.K. (2020). Microbial Biotransformation of Hexavalent Chromium [Cr(VI)] in Tannery Wastewater. Microbial Bioremediation & Biodegradation.

[64] Ackerley D.F., Gonzalez C.F., Keyhan M., Blake R., Matin A. (2004). Mechanism of Chromate Reduction by the *Escherichia coli* Protein, NfsA, and the Role of Different Chromate Reductases in Minimizing Oxidative Stress during Chromate Reduction. Environ. Microbiol..

[65] Ackerley D.F., Gonzalez C.F., Park C.H., Blake R., Keyhan M., Matin A. (2004). Chromate-Reducing Properties of Soluble Flavoproteins from *Pseudomonas putida* and *Escherichia coli*. Appl. Environ. Microbiol..

[66] Han H., Ling Z., Zhou T., Xu R., He Y., Liu P., Li X. (2017). Copper (II) Binding of NAD(P)H- Flavin Oxidoreductase (NfoR) Enhances Its Cr (VI)-Reducing Ability. Sci. Rep..

[67] Shi X.L., Dalal N.S. (1990). NADPH-Dependent Flavoenzymes Catalyze One Electron Reduction of Metal Ions and Molecular Oxygen and Generate Hydroxyl Radicals. FEBS Lett..

[68] Hui C.-Y., Ma B.-C., Wang Y.-Q., Yang X.-Q., Cai J.-M. (2023). Designed Bacteria Based on Natural Pbr Operons for Detecting and Detoxifying Environmental Lead: A Mini-Review. Ecotoxicol. Environ. Saf..

[69] Naik M.M., Dubey S.K. (2013). Lead Resistant Bacteria: Lead Resistance Mechanisms, Their Applications in Lead Bioremediation and Biomonitoring. Ecotoxicol. Environ. Saf..

[70] Castañeda-Barba S., Top E.M., Stalder T. (2024). Plasmids, a Molecular Cornerstone of Antimicrobial Resistance in the One Health Era. Nat. Rev. Microbiol..

[71] McEvoy M.M., Thompson A.M.G. (2013). CusCFBA Copper/Silver Efflux System. Encyclopedia of Metalloproteins.

[72] Rensing C., McDevitt S.F. (2013). The Copper Metallome in Prokaryotic Cells.Metallomics and the Cell. Metal Ions in Life Sciences.

[73] Bondarczuk K., Piotrowska-Seget Z. (2013). Molecular Basis of Active Copper Resistance Mechanisms in Gram-Negative Bacteria. Cell Biol. Toxicol..

[74] Solioz M., Abicht H.K., Mermod M., Mancini S. (2010). Response of Gram-Positive Bacteria to Copper Stress. J. Biol. Inorg. Chem..

[75] Solioz M. (2018). Copper Homeostasis in Gram-Positive Bacteria. SpringerBriefs in Molecular Science.

[76] Achternbosch M., Kupsch C., Sardemann G., Bräutigam K.-R. (2009). Cadmium Flows Caused by the Worldwide Production of Primary Zinc Metal. J. Ind. Ecol..

[77] Khan Z., Elahi A., Bukhari D.A., Rehman A. (2022). Cadmium Sources, Toxicity, Resistance and Removal by Microorganisms-A Potential Strategy for Cadmium Eradication. J. Saudi Chem. Soc..

[78] Alvarez-Ortega C., Olivares J., Martínez J.L. (2013). RND Multidrug Efflux Pumps: What Are They Good For?. Front. Microbiol..

[79] Liu H., Zhang Y., Wang Y., Xie X., Shi Q. (2021). The Connection between Czc and Cad Systems Involved in Cadmium Resistance in *Pseudomonas putida*. Int. J. Mol. Sci..

[80] Abbas S.Z., Rafatullah M., Hossain K., Ismail N., Tajarudin H.A., Abdul Khalil H.P.S. (2018). A Review on Mechanism and Future Perspectives of Cadmium-Resistant Bacteria. Int. J. Environ. Sci. Technol..

[81] Hussain S., Khan M., Sheikh T.M.M., Mumtaz M.Z., Chohan T.A., Shamim S., Liu Y. (2022). Zinc Essentiality, Toxicity, and Its Bacterial Bioremediation: A Comprehensive Insight. Front. Microbiol..

[82] Kandari D., Joshi H., Bhatnagar R. (2021). Zur: Zinc-Sensing Transcriptional Regulator in a Diverse Set of Bacterial Species. Pathogens.

[83] Kambe T. (2012). Molecular Architecture and Function of ZnT Transporters. Curr. Top. Membr..

[84] Bui H.B., Inaba K. (2024). Structures, Mechanisms, and Physiological Functions of Zinc Transporters in Different Biological Kingdoms. Int. J. Mol. Sci..

[85] Dash H.R., Das S. (2012). Bioremediation of Mercury and the Importance of Bacterial Mer Genes. Int. Biodeterior. Biodegrad..

[86] Barkay T., Miller S.M., Summers A.O. (2003). Bacterial Mercury Resistance from Atoms to Ecosystems. FEMS Microbiol. Rev..

[87] Seiler C., Berendonk T.U. (2012). Heavy Metal Driven Co-Selection of Antibiotic Resistance in Soil and Water Bodies Impacted by Agriculture and Aquaculture. Front. Microbiol..

[88] Adhikary S., Saha J., Dutta P., Pal A. (2024). Bacterial Homeostasis and Tolerance to Potentially Toxic Metals and Metalloids through Diverse Transporters: Metal-Specific Insights. Geomicrobiol. J..

[89] Chandrangsu P., Rensing C., Helmann J.D. (2017). Metal Homeostasis and Resistance in Bacteria. Nat. Rev. Microbiol..

[90] Gillieatt B.F., Coleman N.V. (2024). Unravelling the Mechanisms of Antibiotic and Heavy Metal Resistance Co-Selection in Environmental Bacteria. FEMS Microbiol. Rev..

[91] Larsson D.G.J., Flach C.-F. (2022). Antibiotic Resistance in the Environment. Nat. Rev. Microbiol..

[92] Maurya A.P., Rajkumari J., Bhattacharjee A., Pandey P. (2020). Development, Spread and Persistence of Antibiotic Resistance Genes (ARGs) in the Soil Microbiomes through Co-Selection. Rev. Environ. Health.

[93] Nguyen T.H.T., Nguyen H.D., Le M.H., Nguyen T.T.H., Nguyen T.D., Nguyen D.L., Nguyen Q.H., Nguyen T.K.O., Michalet S., Dijoux-Franca M.-G. (2023). Efflux Pump Inhibitors in Controlling Antibiotic Resistance: Outlook under a Heavy Metal Contamination Context. Molecules.

[94] Rillig M.C., Li C., Rodríguez Del Río Á., Zhu Y.-G., Jin L. (2024). Elevated Levels of Antibiotic Resistance Genes as a Factor of Human-Caused Global Environmental Change. Glob. Change Biol..

[95] Carvalho R., Aburjaile F., Canario M., Nascimento A.M.A., Chartone-Souza E., de Jesus L., Zamyatnin A.A., Brenig B., Barh D., Ghosh P. (2020). Genomic Characterization of Multidrug-Resistant *Escherichia coli* BH100 Sub-Strains. Front. Microbiol..

[96] Tuffin I.M., de Groot P., Deane S.M., Rawlings D.E. (2005). An Unusual Tn21-like Transposon Containing an Ars Operon Is Present in Highly Arsenic-Resistant Strains of the Biomining Bacterium Acidithiobacillus Caldus. Microbiology.

[97] Mergeay M., Van Houdt R. (2021). Cupriavidus Metallidurans CH34, a Historical Perspective on Its Discovery, Characterization and Metal Resistance. FEMS Microbiol. Ecol..

[98] Yang S., Deng W., Liu S., Yu X., Mustafa G.R., Chen S., He L., Ao X., Yang Y., Zhou K. (2020). Presence of Heavy Metal Resistance Genes in *Escherichia coli* and *Salmonella* Isolates and Analysis of Resistance Gene Structure in *E. coli* E308. J. Glob. Antimicrob. Resist..

[99] Urquhart A.S., Chong N.F., Yang Y., Idnurm A. (2022). A Large Transposable Element Mediates Metal Resistance in the Fungus *Paecilomyces variotii*. Curr. Biol..

[100] Bukowski M., Piwowarczyk R., Madry A., Zagorski-Przybylo R., Hydzik M., Wladyka B. (2019). Prevalence of Antibiotic and Heavy Metal Resistance Determinants and Virulence-Related Genetic Elements in Plasmids of *Staphylococcus aureus*. Front. Microbiol..

[101] Galetti R., Penha Filho R.A.C., Ferreira J.C., Varani A.M., Darini A.L.C. (2019). Antibiotic Resistance and Heavy Metal Tolerance Plasmids: The Antimicrobial Bulletproof Properties of *Escherichia fergusonii* Isolated from Poultry. Infect. Drug Resist..

[102] Parsons C., Lee S., Kathariou S. (2020). Dissemination and Conservation of Cadmium and Arsenic Resistance Determinants in *Listeria* and Other Gram-Positive Bacteria. Mol. Microbiol..

[103] Souza S.S.R., Turcotte M.R., Li J., Zhang X., Wolfe K.L., Gao F., Benton C.S., Andam C.P. (2022). Population Analysis of Heavy Metal and Biocide Resistance Genes in *Salmonella enterica* from Human Clinical Cases in New Hampshire, United States. Front. Microbiol..

[104] Mei L., Song Y., Liu D., Li Y., Liu L., Yu K., Jiang M., Wang D., Wei Q. (2023). Characterization of a Mobilizable Megaplasmid Carrying Multiple Resistance Genes from a Clinical Isolate of *Pseudomonas aeruginosa*. Front. Microbiol..

[105] Rankin D.J., Rocha E.P.C., Brown S.P. (2011). What Traits Are Carried on Mobile Genetic Elements, and Why?. Heredity.

[106] Feng G., Huang H., Chen Y. (2021). Effects of Emerging Pollutants on the Occurrence and Transfer of Antibiotic Resistance Genes: A Review. J. Hazard. Mater..

[107] Bahram M., Hildebrand F., Forslund S.K., Anderson J.L., Soudzilovskaia N.A., Bodegom P.M., Bengtsson-Palme J., Anslan S., Coelho L.P., Harend H. (2018). Structure and Function of the Global Topsoil Microbiome. Nature.

[108] Apreja M., Sharma A., Balda S., Kataria K., Capalash N., Sharma P. (2022). Antibiotic Residues in Environment: Antimicrobial Resistance Development, Ecological Risks, and Bioremediation. Environ. Sci. Pollut. Res. Int..

[109] Li L., Meng D., Yin H., Zhang T., Liu Y. (2023). Genome-Resolved Metagenomics Provides Insights into the Ecological Roles of the Keystone Taxa in Heavy-Metal-Contaminated Soils. Front. Microbiol..

[110] Zhang H., Huang J., Zeng W., Xiao Q., Zhu Y., Kong W., Zou J., Liu T., Yin H. (2023). Dissecting the Metal Resistance Genes Contributed by Virome from Mining-Affected Metal Contaminated Soils. Front. Environ. Sci..

[111] Khatoon Z., Orozco-Mosqueda M.D.C., Santoyo G. (2024). Microbial Contributions to Heavy Metal Phytoremediation in Agricultural Soils: A Review. Microorganisms.

[112] Shoumik B.A.A., Khan M.Z., Mahmud U., Sultan M.T., Baloch M.Y.J. (2024). Bioremediation of Heavy Metals in Soil by Rhizobacteria for Sustainable Agriculture and Food Security. Bio-Organic Amendments for Heavy Metal Remediation.

[113] Saharan B.S., Chaudhary T., Mandal B.S., Kumar D., Kumar R., Sadh P.K., Duhan J.S. (2023). Microbe-Plant Interactions Targeting Metal Stress: New Dimensions for Bioremediation Applications. J. Xenobiot..

[114] Laborda P., Sanz-García F., Ochoa-Sánchez L.E., Gil-Gil T., Hernando-Amado S., Martínez J.L. (2022). Wildlife and Antibiotic Resistance. Front. Cell. Infect. Microbiol..

[115] Sun H., Li H., Zhang X., Liu Y., Chen H., Zheng L., Zhai Y., Zheng H. (2023). The Honeybee Gut Resistome and Its Role in Antibiotic Resistance Dissemination. Integr. Zool..

[116] Chen C., Li J., Chen P., Ding R., Zhang P., Li X. (2014). Occurrence of Antibiotics and Antibiotic Resistances in Soils from Wastewater Irrigation Areas in Beijing and Tianjin, China. Environ. Pollut..

[117] Zhang R., Yang S., An Y., Wang Y., Lei Y., Song L. (2022). Antibiotics and Antibiotic Resistance Genes in Landfills: A Review. Sci. Total Environ..

[118] Sanganyado E., Gwenzi W. (2019). Antibiotic Resistance in Drinking Water Systems: Occurrence, Removal, and Human Health Risks. Sci. Total Environ..

[119] Argudín M.A., Hoefer A., Butaye P. (2019). Heavy Metal Resistance in Bacteria from Animals. Res. Veterin. Sci..

[120] Karwowska E. (2024). Antibiotic Resistance in the Farming Environment. Appl. Sci..

[121] Hynninen A., Touzé T., Pitkänen L., Mengin-Lecreulx D., Virta M. (2009). An Efflux Transporter PbrA and a Phosphatase PbrB Cooperate in a Lead-Resistance Mechanism in Bacteria. Mol. Microbiol..

[122] van Boeckel T., Pires J., Silvester R., Zhao C., Song J., Criscuolo N., Gilbert M., Bonhoeffer S., Laxminarayan R. (2020). Global Trends in Antimicrobial Resistance in Animals in Low- and Middle-Income Countries. Int. J. Infect. Dis..

[123] Hovorukha V., Moliszewska E., Havryliuk O., Bida I., Tashyrev O. (2024). Metal Resistance of Microorganisms as a Crucial Factor for Their Homeostasis and Sustainable Environment. Sustainability.

[124] Sazykin I., Khmelevtsova L., Azhogina T., Sazykina M. (2023). Heavy Metals Influence on the Bacterial Community of Soils: A Review. Agriculture.

[125] Oyetibo G.O., Enahoro J.A., Ikwubuzo C.A., Ukwuoma C.S. (2021). Microbiome of Highly Polluted Coal Mine Drainage from Onyeama, Nigeria, and Its Potential for Sequestrating Toxic Heavy Metals. Sci. Rep..

[126] Thavamani P., Samkumar R.A., Satheesh V., Subashchandrabose S.R., Ramadass K., Naidu R., Venkateswarlu K., Megharaj M. (2017). Microbes from Mined Sites: Harnessing Their Potential for Reclamation of Derelict Mine Sites. Environ. Pollut..

[127] Cason C., D’Accolti M., Soffritti I., Mazzacane S., Comar M., Caselli E. (2022). Next-Generation Sequencing and PCR Technologies in Monitoring the Hospital Microbiome and Its Drug Resistance. Front. Microbiol..

[128] Bazzi W., Abou Fayad A.G., Nasser A., Haraoui L.-P., Dewachi O., Abou-Sitta G., Nguyen V.-K., Abara A., Karah N., Landecker H. (2020). Heavy Metal Toxicity in Armed Conflicts Potentiates AMR in A. Baumannii by Selecting for Antibiotic and Heavy Metal Co-Resistance Mechanisms. Front. Microbiol..

[129] Eggers S., Safdar N., Kates A., Sethi A.K., Peppard P.E., Kanarek M.S., Malecki K.M.C. (2021). Urinary Lead Level and Colonization by Antibiotic Resistant Bacteria: Evidence from a Population-Based Study. Environ. Epidemiol..

